# The Transcultural Diabetes Nutrition Algorithm: A Middle Eastern Version

**DOI:** 10.3389/fnut.2022.899393

**Published:** 2022-06-13

**Authors:** Osama Hamdy, Saud Al Sifri, Mohamed Hassanein, Mohammed Al Dawish, Raed A. Al-Dahash, Fatheya Alawadi, Nadim Jarrah, Hajar Ballout, Refaat Hegazi, Ahmed Amin, Jeffrey I. Mechanick

**Affiliations:** ^1^Harvard Medical School, Joslin Diabetes Center, Boston, MA, United States; ^2^Alhada Armed Forces Hospital, Taif, Saudi Arabia; ^3^Dubai Hospital, Dubai, United Arab Emirates; ^4^Prince Sultan Military Medical City, Riyadh, Saudi Arabia; ^5^Department of Medicine, Ministry of National Guard-Health Affairs, Riyadh, Saudi Arabia; ^6^King Abdullah International Medical Research Center (KAIMRC), Riyadh, Saudi Arabia; ^7^King Saud bin Abdulaziz for Health Science, Riyadh, Saudi Arabia; ^8^Endocrine Department, Dubai Hospital, Dubai Health Authority, Dubai, United Arab Emirates; ^9^The Specialty Hospital, Amman, Jordan; ^10^Medical University Hospital, Beirut, Lebanon; ^11^Abbott Laboratories, Nutrition Division, Research & Development Department, Columbus, OH, United States; ^12^Abbott Laboratories, Dubai, United Arab Emirates; ^13^Division of Endocrinology, Diabetes and Bone Disease, Icahn School of Medicine at Mount Sinai, Kravis Center for Clinical Cardiovascular Health at Mount Sinai Heart, New York, NY, United States

**Keywords:** diabetes, prediabetes, obesity, nutrition therapy, physical exercise, algorithm, transcultural, Middle East

## Abstract

Diabetes prevalence is on the rise in the Middle East. In countries of the Gulf region—Bahrain, Kuwait, Oman, Qatar, Saudi Arabia, and the United Arab Emirates—prevalence rates are among the highest in the world. Further, Egypt now ranks as one of the top 10 countries in the world for high number of people with diabetes. Medical nutrition therapy is key to optimal management of diabetes. Patient adherence to nutritional guidance depends on advice that is tailored to regional foods and cultural practices. In 2012, international experts created a transcultural Diabetes Nutrition Algorithm (tDNA) for broad applicability. The objective of this current project was to adapt the algorithm and supportive materials to the Middle East region. A Task Force of regional and global experts in the fields of diabetes, obesity, and metabolic disorders met to achieve consensus on Middle East-specific adaptations to the tDNA. Recommendations, position statements, figures, and tables are presented here, representing conclusions of the tDNA-Middle Eastern (tDNA-ME) Task Force. Educational materials can be used to help healthcare professionals optimize nutritional care for patients with type 2 diabetes. The tDNA-ME version provides evidence-based guidance on how to meet patients' nutritional needs while following customs of people living in the Middle Eastern region.

## Introduction

Diabetes is a serious threat to public health around the world, affecting an estimated 10.5% of adults, or 537 million individuals (aged 20–79 years) (1). Compared to 151 million people with diabetes in 2000, this number has more than tripled over the past 2 decades (1). People with diabetes are at risk of cardiovascular and neurological complications. Such health complications reduce patients' quality of life and add to economic burdens for families and nations. Direct costs of health expenditures on diabetes are substantial—an estimated USD$ 966 billion in 2021 (1, 2).

The International Diabetes Federation (IDF) estimated diabetes prevalence in the Middle Eastern-North African region (MENA) at 16.2% in 2021, the highest of any world region (1). In Middle Eastern (ME) Gulf countries (Bahrain, Kuwait, Oman, Qatar, Saudi Arabia [KSA], and the United Arab Emirates [UAE]), prevalence rates ranged from a low of 9.0% in Bahrain to a high of 25.5% in Kuwait. In non-Gulf ME countries, prevalence rates ranged from a low of 8.9% in Lebanon to a high of 18.4% in Egypt (1). In countries like Egypt, the rapid increase in the number of people with diabetes over a relatively short time is alarming; diabetes prevalence in Egypt rose from 4.4 million in 2007–7.5 million in 2013 and is expected jump to 13.1 million by 2035 (3–7). Obesity is a key risk factor for diabetes; over the past 40 years, Egypt and countries of the Gulf region have experienced an obesity epidemic that is largely attributed to urbanization (5–7). This socioeconomic change has been associated with increasingly sedentary lifestyles (5, 6, 8) and to a shift from a traditional ME diet (high fiber, low-fat) to a contemporary diet that is higher in meat, saturated fats, starchy carbohydrates, and sugars, and is lower in fruits and vegetables (3–7).

Diabetes is one of the top 10 causes of death in the world and is associated with more than 10% of deaths globally (1). Almost half of these deaths were of people in the working age group (20 to 60 years of age). Using IDF data for the MENA region, it is estimated that diabetes is responsible for 796,000 deaths annually in adults ages 20 to 79, including the highest proportion of deaths due to diabetes in people under 60 years of age (24.5%) of any world region (1).

Medical nutrition therapy (MNT) and regular physical activity are essential to optimal management of prediabetes and type 2 diabetes (T2D) (9–13). However, the half billion people affected by these conditions worldwide encompass a vast range of cultural backgrounds, personal dietary preferences, socioeconomic conditions, and comorbidities. As such, experts recognize that patients' needs differ around the world and that adherence to nutritional guidance can be enhanced when recommendations are tailored to individual requirements, personal habits, and cultural factors (11, 14).

International experts in fields of nutrition, obesity, diabetes, and metabolic disorders thus created the transcultural Diabetes Nutrition Algorithm (tDNA) (15). The tDNA represents a template for country-specific or regional adaptation, thus facilitating greater precision to recommendations by healthcare professionals (15). The initial tDNA template has been adapted for nations around the world (16–19). This paper presents the tDNA Middle Eastern (ME; tDNA-ME) version, which was based on an interactive collaboration of international and Middle Eastern experts in the fields of diabetes, obesity, and metabolic disorders. The tDNA-ME represents nutritional guidance for people with diabetes in Gulf (Bahrain, Kuwait, Oman, Qatar, KSA, and the UAE) and non-Gulf countries (Egypt, Jordan, Lebanon) of the Middle East.

## Methods

### Expert Recommendation Process

The first meeting date of the ME Task Force was held in January 2019, and several electronic follow-up meetings took place in 2020. The ME Task Force built region-specific recommendation based on the tDNA template. Here the nutritional guidance was specifically adapted to the ME geographic region. Given that majority of the ME population is Muslim (93%) (20) and that extended daytime fasting significantly influences glycemic control, we include Ramadan fasting as a key transcultural factor to address. All recommendations were approved by a vote from each participating member. All Task Force members who are authors specifically approved the content of this summary manuscript. Authors are shown here with their specialties: Osama Hamdy (diabetes international); Saud Al Sifri (diabetes Middle East); Mohamed Hassanein (diabetes and endocrinology Middle East); Mohammed Al Dawish (diabetes and endocrinology Middle East); Raed A. Al-Dahash (diabetes and endocrinology Middle East); Fatheya Alawadi (diabetes Middle East); Nadim Jarrah (diabetes and endocrinology Middle East); Hajar Ballout (endocrinology Middle East); Refaat Hegazi (diabetes international, nutrition international); Ahmed Amin (nutrition Middle East); and Jeffrey I. Mechanick (diabetes international, endocrinology, metabolism).

### Transcultural Diabetes Nutrition Algorithm-ME

**Figure 1:**
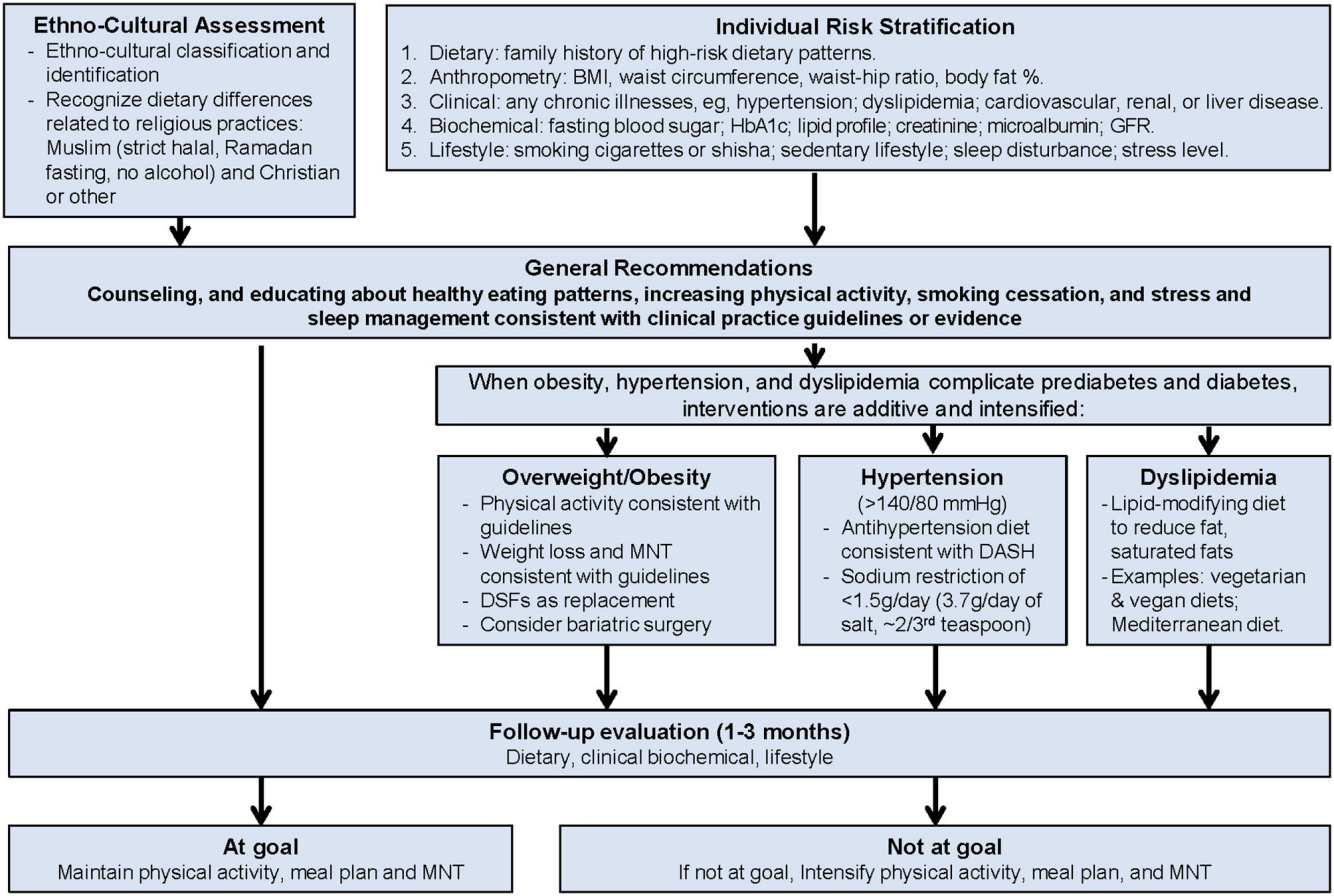
Middle East transcultural diabetes nutrition algorithm for prediabetes and type 2 diabetes. Figure adapted for the ME from Mechanick et al. (15).

The objective for the ME Task Force was to adapt the initial Transcultural Diabetes Nutrition Algorithm (tDNA) for T2D to meet ethno-cultural needs of the ME region (Figure 1). Changes acknowledge differences in food availability and preferences, as well as cultural practices that affect diet. The algorithm is relevant to people with diabetes who are living in the Middle East and eating locally available foods, including those of all religions and ethnicities. For the Muslim majority, we have added dietary guidance for managing glycemic status for those who fast during the month of Ramadan.

## Middle Eastern Transcultural Factors

Various risk factors account for the rapid rise in diabetes in the Middle, including environmental and behavioral factors along with genetic factors (21). Strategies such as clinical counseling for changes in dietary habits and increases in physical activity, along with medical treatment of hypertension and dyslipidemia, can be used to lower risks associated with T2D/overweight. For some, fasting during Ramadan, infrequent intermittent fasting throughout the year, and drinking diabetes-specific formulas (DSFs) as meal or snack replacements are strategies that can help control glycemic status and manage weight, thus lowering risk for poor health outcomes.

### Changing Dietary Patterns

A dramatic transition from the traditional ME diet to a more Western diet is widely attributed to rapid urbanization over the past 40 years (5, 6, 14, 22, 23). The traditional ME diet is high in fiber and low in fat. Since the 1970's, however, regional diets have shifted dramatically with rising incomes and more Western and urban lifestyles, to consumption of more meat, fast foods, and processed food. As result, diets in the region are higher in calories from starchy carbohydrates, added sugar, and saturated fat (mostly from animal origin) but lower in complex carbohydrates, dietary fiber, fruit, and vegetables (5, 6, 14). Recent studies in young people of the region affirm the persistent appeal of fast food, sweetened beverages, and processed and fried foods (22–24); over 55% of Saudi university students who were overweight or obese consumed fast food more than three times per week, and nearly 50% consumed carbonated/flavored drinks or energy drinks just as frequently (22, 23). Furthermore, at least 70% of all students reported eating fiber-rich fruits and vegetables *only rarely* or one *to two times per week* (23). Sodium consumption is also relatively high in the Gulf region, as people commonly prefer salty foods. The natural salinity of drinking water further contributes to high sodium intake (14).

### Physical Activity and Sedentary Lifestyles

Physical activity and structured exercise have been shown to improve blood glucose control, reduce cardiovascular risk factors, and contribute to weight loss (25). Moderate-to-high volumes of aerobic activity are associated with substantially lower cardiovascular and overall mortality risks in both type 1 diabetes and T2D (26). Conversely, inactivity is one of the leading risk factors for noncommunicable diseases and death worldwide, increasing the risk of diabetes, cancer, heart disease, and stroke by 20–30% (27).

The shift to higher incomes and urban living resulted in highly sedentary lifestyles, especially in the Gulf region of the ME. Studies from the first decade of the 2000s found rates of physical inactivity in the Gulf were among the highest in the world (6). More recent studies reveal that inactivity remains a serious issue for people of all ages; 65–88% of youth and 40–96% of adults are considered inactive (28). A recent review of obesity studies found that lack of physical activity was one of the most consistent correlates of overweight/obesity in the Gulf States, and most studies showed women had lower levels of physical activity than men (5).

Some barriers to activity are common to populations in other developed countries: lack of time, motivation, and facilities (28). Other issues are particular to this region, such as a hot climate that limits outdoor activity; inadequate availability of indoor facilities to compensate; and a lack of encouragement from families and peers resulting from a culture that deemphasizes physical activity (21, 28). Some gender constraints in the Gulf region make it difficult for women to participate in physical activity, e.g., conservative dress, few gender-segregated facilities (28).

### Overweight and Obesity

There is substantial evidence linking obesity/overweight to diabetes and indicating that weight loss is highly effective in preventing progression from prediabetes to T2D and in managing cardiometabolic health in people with T2D (11). Within this context, obesity/overweight can be identified on the basis of body mass index (BMI) cutoffs, or it can be observed as part of a cluster of metabolic conditions known as “metabolic syndrome” (MetS), or as stage 3 *adiposity-based chronic disease*, one of several metabolic drivers of cardiometabolic-based chronic disease (CMBCD) (29).

The prevalence of obesity and overweight (BMI ≥25 kg/m^2^) in the Gulf States has risen markedly in the last four decades. The WHO Global Health Observatory estimated the prevalence among adults ranging from a low of 63% in Oman to a high of 72% in Qatar (30). More women than men are overweight or obese by several percentage points in each nation. From 27% (Oman) to 38% (Kuwait) of all adults are obese (BMI ≥30 kg/m^2^). However, many more women than men are obese in all countries in the region. For example, while 33% of men are obese in both Kuwait and Qatar, 43% and 46% of women are obese in those countries, respectively (30). In Egypt, 49% of adult women are obese in a recent national health survey. The first national health survey in Kuwait found that 48% of males with obesity and 77% of females with obesity also had diabetes, confirming a significant association between obesity and diabetes in the Kuwaiti population (21). In Oman and Qatar, approximately 60% of the people with diabetes were also obese (21).

### Hypertension and Dyslipidemia

When obesity, hypertension, and dyslipidemia complicate prediabetes and diabetes, health risk increases (31) and interventions are added and intensified, as noted in the T2D treatment algorithm (Figure 1). People with T2D have a significantly increased risk of atherosclerotic cardiovascular disease (ASCVD), compared to those without diabetes (31). Controlling blood glucose is fundamental to preventing microvascular complications of diabetes, and managing dyslipidemia is fundamental to preventing macrovascular disease, or ASCVD (31).

For the ME, data are scant about the concomitant occurrence of hypertension and dyslipidemia in people with diabetes. However, the World Health Organization (WHO) reports that hypertension is common. Specifically, an estimated 20% to 25% of adults living in Gulf region states have systolic blood pressure ≥ 140 mmHg (30). While dyslipidemia for people with diabetes is defined in terms of triglycerides and high-density-lipoprotein cholesterol (HDL-C) levels and a procoagulant and pro-inflammatory milieu (31), the WHO uses a single criterion, i.e., total cholesterol ≥ 6.2 mmol/L. Based on the WHO definition, an estimated 10% (KSA) to 17% (Qatar) of adults are living with dyslipidemia (30).

### Ramadan Fasting

Fasting during Ramadan is foundational to Islam and is obligatory for healthy adult Muslims. The onset of Ramadan requires a sudden shift in mealtimes for those who are fasting. In some years, Ramadan can occur in hot and dry months and during extended daylight hours, requiring longer fasting intervals (32). For people with diabetes (with varying degrees of insulin resistance and insulin deficiency), fasting can lead to higher than usual levels of glycogen breakdown, gluconeogenesis, and/or fat breakdown. As a result, hypoglycemia, hyperglycemia, diabetic ketoacidosis, dehydration, and thrombosis risks are all increased (32). Despite such risks, many Muslims with diabetes choose to fast. Using expert guidelines for care of people with diabetes during Ramadan, healthcare professionals can advise their patients about how to avoid these serious metabolic complications (32).

### Role of Medical Nutrition Therapy in Treatment of Diabetes

When T2D is identified, MNT plays an integral role in management of T2D in the ME region, as elsewhere. MNT includes nutritional assessment, diagnosis, intervention, and monitoring provided by a nutrition professional. Notably, many people with diabetes have difficulty determining what to eat when self-managing their diabetes condition. In response, experts call for individualized eating plans developed in conjunction with a patient's healthcare team and registered dietitian nutritionist (11, 26). The benefits of MNT on glycemic control in people with prediabetes and T2D have been well established (10, 12), and the cost-effectiveness of MNT has likewise been demonstrated (11). However, very few trials of lifestyle interventions among people with diabetes have been undertaken in the Gulf States, and those that are available are of questionable quality (33). Available studies indicate that education and counseling can lead to improved glycemic control (with nutrition counseling) and to weight loss (with physical activity programs) (33).

### DSFs for People With Diabetes and Other Metabolic Disorders

Specialized nutritional supplements (diabetes-specific formulas, DSFs) have been designed specifically for use by people with diabetes as a meal replacement or as a snack. The American Diabetes Association (ADA) recommends meal replacements as a safe and effective way to help achieve weight loss (9). DSFs are helpful for meal planning because they are convenient and provide a known calorie and macro- and micronutrient profile (34). Replacing a typical breakfast with DSFs favorably impacted postprandial glycemic responses and replacing an afternoon snack by DSFs reduced overnight glucose variability in participants (34). In a systematic review comparing DSFs with standard formulas, results showed that DSF use consistently resulted in significantly lower postprandial rise in blood glucose, lower peak blood glucose concentrations, and smaller glucose area under the curve (AUC) in patients with diabetes (35). Such results were achieved without evidence of hypoglycemia (35).

## Specific Recommendations and Discussion

The following recommendations, statements and tables represent the conclusions of the tDNA-ME Task Force, accommodating regional differences in lifestyle, foods, and customs to meet the needs and preferences of people with T2D in the ME.

### ME Recommendation 1–Review Diet of Patients With Non-communicable Diseases and Identify Specific Elements That Are Unhealthy and Can Be Improved in a Culturally Sensitive Way

Eating patterns and diet composition influence the risks of developing chronic non-communicable diseases, such as obesity, prediabetes, and T2D. Ask about dietary patterns and specific food choices when interviewing such patients in routine healthcare visits. Recognize that although most people living in the ME are Muslim, the region includes people of other ethnicities, cultures, and religions.

### ME Recommendation 2–Assess Body Composition, Metabolic Markers, and Determine Risk for CMBCD Progression

All patients should be evaluated with a full clinical history and a complete physical examination focused on nutritional status, fat distribution, dysglycemia, other CMBCD markers, and comorbidities (e.g., BMI, waist circumference, fasting and post-challenge plasma glucose, A1C, blood pressure, and lipid profile). At the end of the consultation, a risk assessment should be determined based on measures of plasma glucose, hemoglobin A1c, and body weight (Tables 1, 2).

**Table 1:** Diagnostic criteria for prediabetes and diabetes*.

	**Prediabetes**	**Diabetes**
FPG (mg/dl)	100–125	≥126
2h OGTT (mg/dl)	140–199	≥200
Casual PG (mg/dl)	<200	≥200
A1C (%)	5.7–6.4	≥6.5

*A1C, hemoglobin A1c; FBS, fasting blood sugar; IGT, impaired glucose tolerance; PG, plasma glucose; 2h OGTT, 2-h oral glucose tolerance test. See reference (36).

**Table 2:** Effects of body composition on cardiometabolic risk in the Middle East*.

			**Waist Circumference**	
			and Disease Risk*	
	**BMI, kg/m^**2**^**	**Obesity class**	**Men ≤ 102cm Women ≤88 cm**	**Men > 102 cm** **Women > 88 cm**
Underweight	<18.5			
Normal weight	18.5–24.9			
Overweight	25.0–29.9		Increased	High
Obese	30.0–34.9	I	High	Very high
	35.0–39.9	II	Very high	Very high
Extremely obese	≥40	III	Extremely high	Extremely high

*Risk levels for cardiometabolic drivers and outcomes (T2D, hypertension, and CVD) are determined by the combined effects of BMI and WC. Note that BMI and WC cutoffs are like those for Caucasians in other regions. BMI, body mass index; WC, waist circumference. See reference (37–40).

### ME Recommendation 3–Incorporate Culturally Adapted MNT in the Comprehensive Management of T2D

MNT is crucial in the prevention and treatment of T2D and other non-communicable chronic diseases and should be recommended for all patients, always respecting their social, cultural, and economic circumstances.

### ME Recommendation 4–Culturally Adapt Lifestyle Interventions in the Routine Management of T2D

Key lifestyle interventions – MNT and regular physical activity – should be customized to meet the unique clinical needs and conditions of individual patients through professional counseling and should be consistent with current clinical practice guidelines and local habits and practices (Supplementary Table 1). Consideration should be made for age, gender, anthropometrics, biomarkers, cardiometabolic drivers, comorbidities, and disabilities (see ME Recommendation 4.5).

Weight management is important for people with overweight or obesity. Those with prediabetes should lose 7–10% of their body weight to prevent progression to T2D (36). For those unable to achieve and sustain a 7–10% weight loss with lifestyle therapy alone (physical activity and improved nutrition), medication-assisted weight loss can be considered.

Monitoring to assess whether nutritional and physical activity goals are being achieved should occur at diabetes diagnosis; then annually for assessment of progress; and when new health complications or changes in care occur (26).

**Figure 2:**
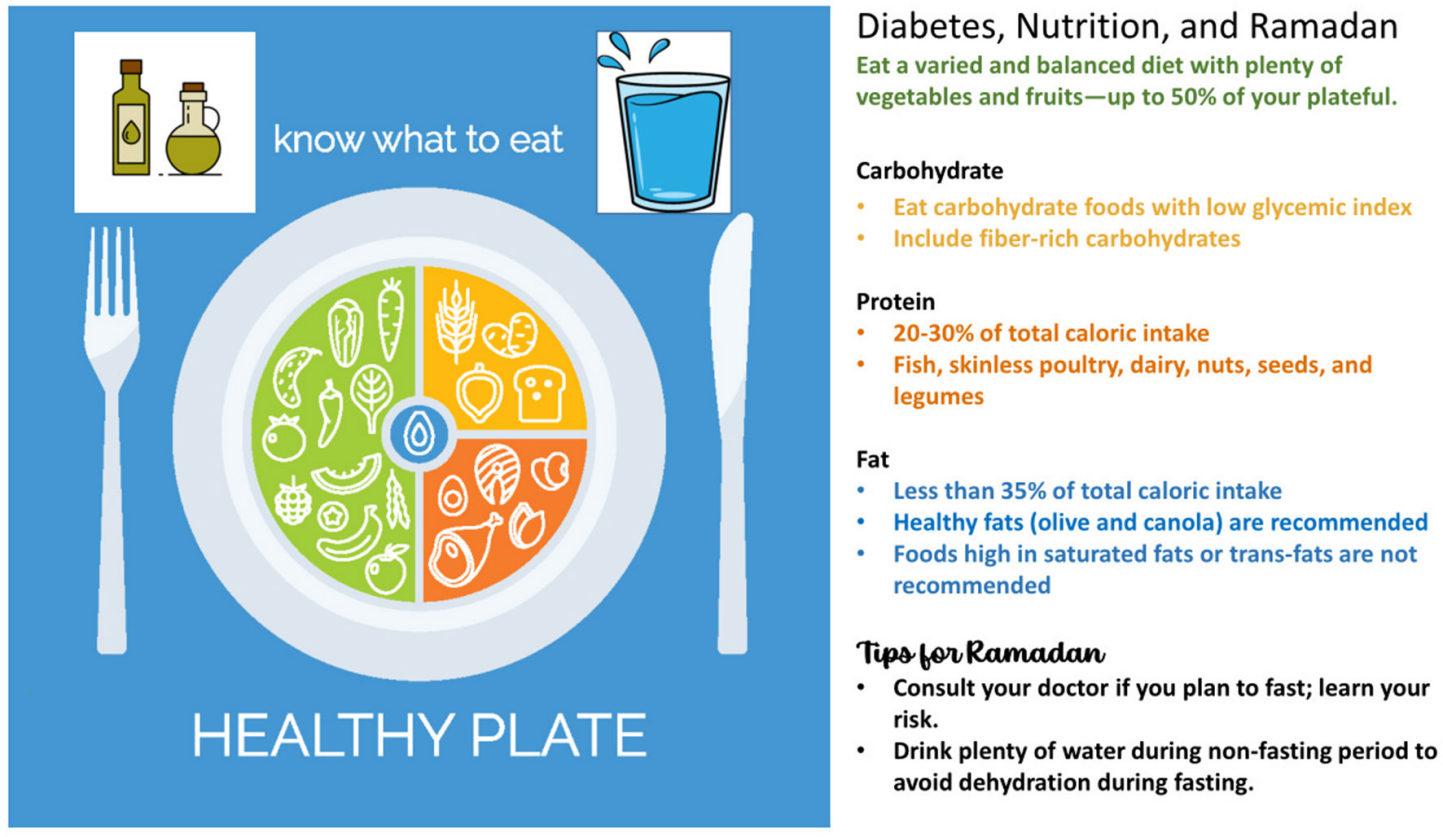
A healthy eating plate to guide dietary intake. Figure information sources (32, 42) with healthy plate image from istockphoto.com.

Evidence suggests that there is no ideal percentage of calories from carbohydrate, protein, and fat for people with diabetes, however lowering carbohydrates intake is the most valuable. Instead, macronutrient distribution should be based on an individualized assessment of current eating patterns, preferences, and metabolic goals (26). Diets should be high in fiber [recommended intake is 14 g of fiber per 1,000 kcals (11, 41)] and consist of minimally processed foods. General guidance is illustrated and summarized in Figure 2.

### ME Recommendation 5–Culturally Adapt Lifestyle Interventions in the Routine Management of Hypertension and Dyslipidemia in Patients With T2D

Lifestyle modifications are also the first line of treatment for hypertension. For patients with blood pressure ≥140/80 mmHg, lifestyle intervention should consist of weight loss if overweight or obese; a Dietary Approaches to Stop Hypertension (DASH)-style eating pattern (Table 3) that includes reducing sodium and increasing potassium intake; consuming a diet rich in whole grains, fruits, vegetables; and reducing intake of sugars, saturated fats and trans fats (43). Regular physical activity and smoking cessation are also recommended (46, 47).

**Table 3:** Antihypertensive DASH dietary goals for the Middle East^*^.

**Nutrient**	**Recommendations**
**Macronutrients**	
Protein	18% of total calories
Carbohydrate	55% of total calories
Fiber	30 g/day
Total fat	27% of total calories
Saturated fat	6% of total calories
Cholesterol	150 mg/day
**Micronutrients**	
Sodium	1500 mg/day
Potassium	4700 mg/day
Calcium	1250 mg/day
Magnesium	500 mg/day

*The above percentages are based on 2,000 kcal/day eating plan. DASH, Dietary Approaches to Stop Hypertension. See references (43–45).

A Mediterranean eating pattern can improve both glycemic management and blood lipids (11, 46). The Mediterranean diet resembles a traditional ME diet, and both the Mediterranean and the traditional ME diets markedly contrast with the urbanized diet of many Middle Easterners today.

Features of a Mediterranean-style diet include:

- Plant-based: abundant in fruits and vegetables; breads and other forms of cereals; and beans, nuts, and seeds.
- Minimally processed food, such as regionally grown, seasonally fresh foods.
- Limited sweets: fresh fruits are the typical daily dessert; otherwise, select sweets based on nuts and made with olive oil.
- High-quality fats: olive oil is the primary source of fat, and total intake is moderate (30%) to high (40%) proportion of total energy intake.
- Moderate dairy intake: eat mainly cheese and yogurt; eat few or no dairy products that have sweeteners added.
- Protein: red meats and eggs are consumed in small amounts and with low frequency; seafood intake varies, with moderate amounts of fish.
- Herbs and spices: use these instead of salt to add flavor to foods.

### ME Recommendation 6–Use Regional Food Preferences in Managing T2D

**Figure 3:**
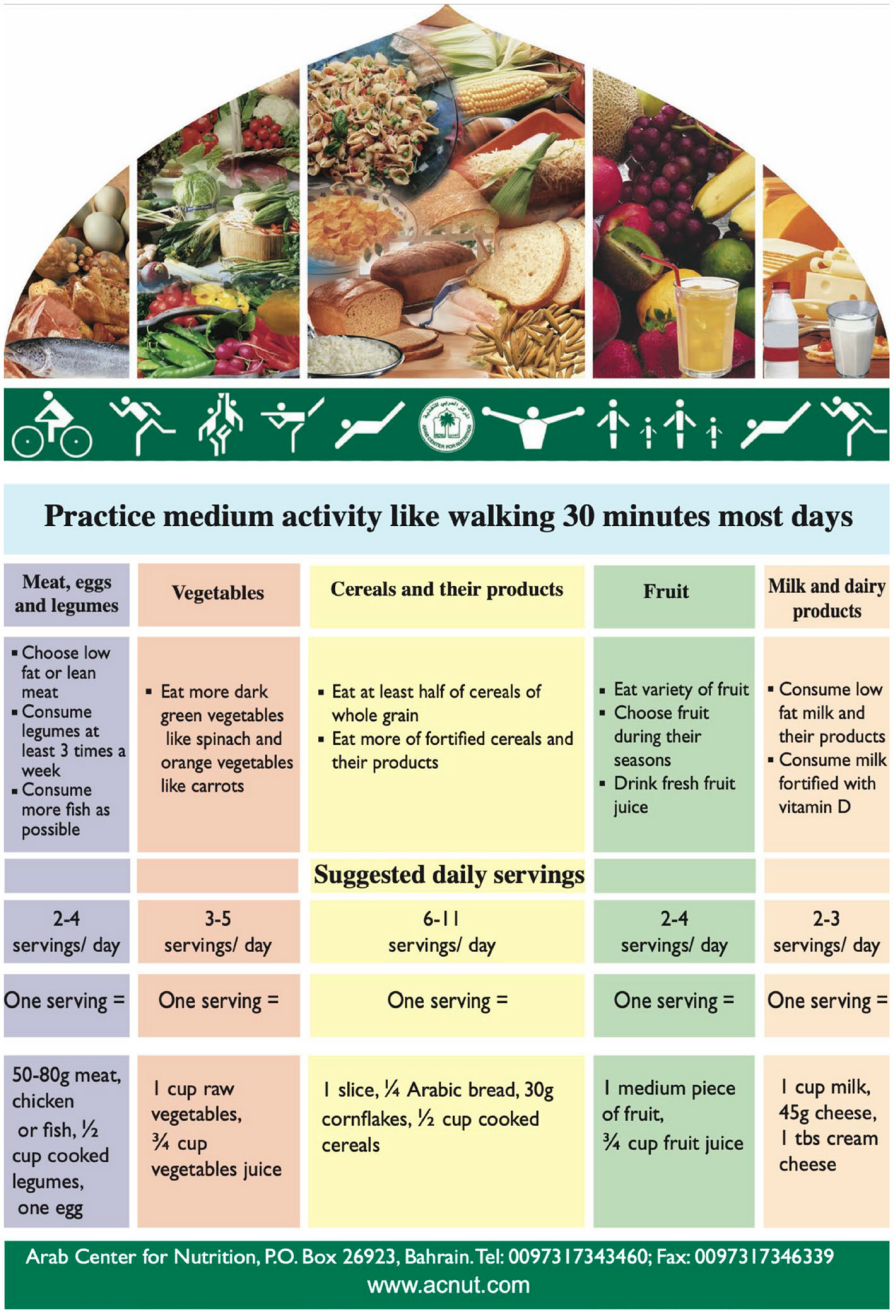
Arab Food Dome. Figure reference (48).

When providing MNT, cultural preferences can guide the selection of regional foods and meals and are consistent with general nutritional recommendations (9, 31). In absence of dietary guidelines specific to people in or from the Middle East, regional health institutes have primarily used Western dietary guidelines. As ME transcultural recommendations become more widely available, ME professional will be increasingly prepared to offer culturally relevant dietary guidance. Notably, the Arab Centers for Nutrition established the “Food Dome” (48), a dietary guideline to prevent disease in the Arab region (Figure 3).

Some common regional food options are listed with their glycemic indices (GI) in Tables 4, 5. The concept of GI provides a way to compare the glycemic response of different foods by indexing how quickly the carbohydrates in a food raise blood glucose relative to a similar weight of a reference food–usually glucose or white bread (52, 53). The foods in Table 4 are classified by food group and listed from low to high GI relative to white bread at a GI of 100. Most fruits, non-starchy vegetables, whole grains, and legumes are low GI foods. Carbohydrate-free proteins such as meats, seafood, and poultry have a zero or very low GI values; as expected, the GI is increased when the protein is cooked in combination with rice or sauce. Table 5 features mixed-ingredient dishes from countries in the Middle East; dishes are listed by GI value from low to high.

**Table 4:** Glycemic index (GI) values of some common Middle Eastern foods by food group*.

**Fruits and vegetables**		**Meat, fish, dairy**		**Carbohydrates**	
**Food**	**GI**	**Food**	**GI**	**Food**	**GI**
Carrots, raw	35	Chickpeas	10	Khameer bread	47
Carrots, boiled	39	Yogurt, plain Greek	11	Multigrain bread	53
Dates, Khalas	36	Feta cheese	27	Chebab bread	54
Apple	38	Lentils	29	Brown rice	55
Arnana	52	Milk, fat-free	32	Oatmeal	58
Grapes	59	Yogurt, sweetened fruit	36	White rice, boiled	64
Sweet corn	60	Milk, full fat	41	Couscous	65
Banana	62	Burghol	48	Arabic pita bread	67
Watermelon	72	Chicken, biryani	52	Popcorn, air popped	72
Dates, Sellaj	75	Fish machboos	60	Fendal, sweet potato	74
		Beef thareed	74	Regag bread	76
		Cheese fatayer	80	Muhalla bread	77
				Tannour white bread	81
				Corn flakes	81
				Awama	81
				White potato, boiled	82
				Sharia (vermicelli)	83
				White basmati rice	84

*The GI formula is (iAUC of a test food divided by iAUC of a reference food) × 100. iAUC, incremental area under curve; GI, glycemic index. See references (3, 49–51).

*GI values are low at < 55; 56–69 values are moderate; and values >70 are high*.

**Table 5:** Glycemic index of common Middle Eastern dishes.

**Food**	**GI**	**Food**	**GI**
Fatayer cheese (Qatar)	80	Stuffed cabbage with rice & meat	67.9
Fatayer zaatar (Qatar)	80	Green beans in oil	12.8
Fatayer spinach (Qatar)	78	Baked muttabaq (Saudi Arabia)	56
Machbous fish (UAE)	60	Harees (Saudi Arabia)	52
Burghol with tomatoes (Lebanon)	50		
Harees (UAE)	42		
Thareed beef (UAE)	74	***Desserts:***	
Biryani chicken (UAE)	52	Awama (fried doughnuts Qatar)	81
Arseyah (basmati rice w/chicken)	72	Qurs Aquili (Qatar)	83
Khabisa (semolina with cardamon)	67	Muhalabia (milk with starch & sugar)	83
Pizza	56	Riz bi halib (milk with rice & sugar)	57
Sambosa vegetable	60	Batheetha (khalas date paste)	59
Red beans with white bread	61	Kanfaroosh (doughnut cake)	45
Mjadara (lentils & rice, Lebanon)	24	Balaleet	63
Stuffed grape leaves	30	Shearia (Qatar)	83
Moroccan couscous	58	Dates with Arabic coffee	63
Kibbeh saynyeh	61	Dates with sour milk or yogurt	29

*The GI formula is (iAUC of a test food divided by iAUC of a reference food) × 100. iAUC, incremental area under curve; GI, glycemic index; UAE, United Arab Emirates. See reference (3, 50, 51)*.

The amount of food eaten must also be considered when estimating glycemic response; a large amount of even a low GI food will significantly increase glycemic response. Thus, the concept of glycemic load (GL) was introduced to better predict a person's glycemic response after consuming a meal or snack (53). Using these figures to make lower GI/GL food choices can improve blood glucose control (52).

### ME Recommendation 7–Manage Ramadan Fasting Using Guidelines for Healthy Eating in Patients With T2D

Ramadan fasting can be risky for many people with T2D. In general, we advise following guidelines for healthy eating (Figure 2). Practical guidelines created specifically to advise management of people with diabetes during Ramadan suggest a visit to a healthcare professional for a risk assessment and advice well before the start of the fast (32). Structured education should be included, covering self-monitoring, when to break the fast (based on blood glucose levels), fluid consumption, meal planning, and medication adjustments (32). Specific strategies for a Ramadan dietary plan include the following (32):

- Divide total daily calories between the 2 meals—suhoor (sunrise meal) and iftar (sunset meal).
- If necessary, consume 1–2 low-calorie snacks between meals (fruit, nuts, or vegetables).
- Design meals for macronutrient balance (45–50% carbohydrate, 20–30% protein and <35% fat).
- Avoid sugar-loaded desserts.
- Preferentially include low-GI and high-fiber carbohydrates.
- Maintain hydration between meals by drinking water and non-sweetened beverages.
- Eat suhoor as late as possible to avoid hypoglycemia during the day.
- Include adequate protein and fat at suhoor to help maintain satiety through the day.
- Begin iftar with water to rehydrate.
- Consume no more than 2 dates at iftar to avoid a rapid rise in blood glucose.

Although people with diabetes and with very high-risk classification are advised to avoid fasting, a Saudi study found that a large majority chose to fast, and one-quarter experienced hypoglycemic episodes directly related to their fasting (54).

### ME Recommendation 8–As Part of a Comprehensive T2D Management Plan, Incorporate Sufficient Physical Activity That Is Consistent With Customs and Practices

All adults, and particularly those with T2D, should increase levels of both incidental and structured physical activity and reduce the amount of time spent in sedentary behavior (Supplementary Table 2). Specific recommendations and precautions will vary by age, activity, and presence of diabetes-related health complications, and should thus be tailored to meet the specific needs of each individual (26). Regular physical activity helps to improve insulin sensitivity and glycemic control, positively affects lipids and blood pressure, assists with weight maintenance, and is associated with reduced risk for cardiovascular disease (55). Prolonged sitting should be interspersed with bouts of light activity every 30 min for blood glucose benefits (56).

Daily exercise, or at least not allowing more than 2 days between exercise sessions, is recommended to help reduce insulin resistance, i.e., enhance the normal actions of insulin and reduce adverse effects on beta-cell function. Adults with T2D should ideally perform at least 150 min per week of both aerobic and progressive resistance exercise training for optimal glycemic and health outcomes. The physical activity should be of moderate exertion (e.g., walking or stair climbing). Those who are obese or have related complications should consider a structured program of physical activity with monitoring. For those with the greatest burden of obesity and complications, physical activity should be undertaken after medical evaluation and preclearance, with ongoing medical supervision (57). Children and adolescents with T2D should be encouraged to meet the same physical activity goals set for youth in general (56).

To prevent or delay the onset of T2D, people at high risk for and with prediabetes should engage in structured lifestyle interventions that include at least 150 min/week of physical activity and dietary changes resulting in weight loss of 5–7% (56). In the Middle East, people with diabetes should take care to hydrate properly and take care on hot, humid days. Hyperglycemia increases risk through dehydration caused by osmotic diuresis, and some medications prescribed to lower blood pressure may also impact hydration and electrolyte balance. In the Middle East, cultural and demographic must be considered when recommending physical activities. For instance, people with T2D may be more amenable to walking indoors rather than outdoors. Advice to walk inside malls and shopping centers may increase adherence to recommendations for increased physical activity.

Older adults with diabetes or anyone with autonomic neuropathy, cardiovascular complications, or pulmonary disease should avoid exercising outdoors on very hot or humid days (56).

### ME Recommendation 9–Consider DSFs and Glucose Monitoring for Glycemic Control and Management of Overweight/Obesity in Patients With T2D

DSF formulas can be used for calorie replacement (9, 58) or for supplementation as part of MNT (15, 34, 59). DSF can be used as partial or full meals or as replacement for snacks restrict caloric intake and support metabolic control in patients who are overweight or obese. For caloric supplementation and metabolic control in patients who are underweight or experience sarcopenia; or for metabolic control in patients with normal weight but elevated glucose levels (Table 6). Glycemic targets should be individualized for each patient based on local clinical practice guidelines, and it is suggested that products meet the ADA nutritional guidelines. However, incorporating DSF into common Middle Eastern eating patterns is essential for adherence. For example, portions of DSF can be used to replace high calorie and saturated fat-dense desserts or snacks.

**Table 6:** DSFs for prediabetes and diabetes^*^.

**BMI classification**	**Commercial DSF Recommendation**
Overweight or obese	• Use 2 to 3 units per day^a^ as part of a reduced calorie meal plan, as a calorie replacement for a meal, partial meal, or a snack. • Daily calorie goals from diabetes-specific nutrition formulas and other healthy dietary sources: Women = 1200 to 1500 calories Men = 1500 to 1800 calories
Normal weight	Uncontrolled diabetes, A1C > 7%	1 to 2 units per day incorporated into a meal plan, as a calorie replacement for a meal, partial meal, or a snack.
	Controlled diabetes, A1C ≤ 7%	Use should be based on individual patient needs and clinical judgment of the healthcare professional^b^
Underweight	1 to 3 units per day per clinical judgment based on desired rate of weight gain and clinical tolerance^c^	

^*^Definitions: DSF, Diabetes-specific formula for nutrition.

^a^*DSFs are complete and balanced products with at least 200 calories per serving used as part of a meal plan to help control calorie intake and achieve glycemic control*.

^b^*Meal and snack replacements are nutritional products used to replace dietary calories*.

^c^*To avoid hypoglycemia or postprandial hyperglycemia, individuals who may have muscle mass and/or function loss and/or micronutrient deficiency may benefit from a nutrition supplement. Individuals who need support with weight maintenance and/or a healthy meal plan could benefit from meal replacement*.

*A1C, hemoglobin A1c; BMI, body mass index; DSF, diabetes specific formula for nutrition*.

*See reference (15)*.

Monitoring blood glucose is important to the overall success of lifestyle interventions. Dietary awareness by monitoring carbohydrate intake, either by counting or by tallying units of exchange, can be used to facilitate glycemic management (60). Further, recent advances in continuous glucose monitoring could potentially guide diet and activity choices for people with diabetes. Real-time monitoring of glucose levels—especially around times of eating, physical activity, sleep, and medication-taking—can help patients make good dietary and exercise choices for optimal glycemic control (61).

### ME Recommendation 10–Consider Bariatric Procedures as Part of a Management Algorithm for Patients With T2D and/or High Adiposity

Bariatric procedures (surgical and nonsurgical) were originally designed to promote weight loss in patients with severe obesity. More recently, these procedures showed efficacy for managing recalcitrant T2D, especially when the surgery included some form of intestinal bypass (62, 63). Underlying mechanisms are diverse and depend not only on weight loss but also on modulation of various physiological pathways (62).

Bariatric procedures can be considered for patients who have attempted lifestyle modification but failed to achieve and sustain weight loss; are expected to tolerate the risk of surgery; are committed to treatment and long-term follow-up; and have accepted the required lifestyle changes. Only limited guidance is presently available on use of bariatric surgery in ME populations. A recent review identified cultural-specific considerations that may affect bariatric care and outcomes in 6 domains: knowledge of bariatric surgery; mental health, body image, and quality of life; influence of family; religion and lifestyle; preoperative practices; and healthcare access (64). Because of the high burden of obesity in the ME, bariatric surgery is common; laparoscopic sleeve gastrectomy was reported to be the most frequently used procedure (65).

General recommendations for the use of bariatric surgery follow (62, 66) (Supplementary Table 3):

- For people with T2D and BMI ≥40 kg/m^2^ (about 100 pounds overweight for men and 80 pounds for women), bariatric surgery is recommended.
- For people with T2D and BMI between 35-39.9 kg/m^2^, and a serious obesity-related comorbidity, such as coronary heart disease, or severe sleep apnea, bariatric surgery is recommended.
- For people with T2D and BMI 30-34.9 kg/m^2^, bariatric surgery can be considered as an alternative treatment option for those with special circumstances, for example when diabetes is not adequately controlled by an optimal medical regimen, especially when there are risks for cardiovascular disease.

## Conclusions

The tDNA-ME version and accompanying lifestyle recommendations presented here result from the cultural adaptation of established evidence-based clinical practice guidelines for the treatment of diabetes, nutritional disorders, obesity, cardiovascular disease, and related lifestyle issues. Issues particular to the cultural environment of the ME are outlined and serve as components of a set of transcultural recommendations related primarily to nutrition and physical activity to reduce the development, progression, and impact of chronic (especially cardiometabolic) disease. For educational and training support, all Tables and Figures of this document have also been compiled in the Supplementary Material.

ME healthcare professionals are encouraged to educate patients with diabetes about the importance of following nutritional and physical activity recommendations. Such changes in lifestyle can help maintain glycemic control, enhance overall health status, and improve long-term outcomes.

## Author Contributions

OH served as chairperson for the group and prepared a summary report. JM served as a senior technical author-reviewer. All authors participated in the Task Force meetings, contributing concepts and content for inclusion in the manuscript, reviewed and edited the manuscript in draft form, and agreed upon the final version.

## Funding

This Middle Eastern Task Force project was funded by Abbott Nutrition MENAP, including meeting costs, honoraria for participating experts, and funds to cover publication costs.

## Conflict of Interest

AA and RH were employees of Abbott Laboratories, the material presented in this article is based on the best-known clinical evidence and is not affected by this financial relationship. JM has received honoraria from Abbott Nutrition, and he serves on Advisory Boards of Aveta, Life, L-Nutra, and Twin Health. OH has received research support from Novo-Nordisk, Eli-Lilly, Gilead, and National Dairy Council, he is on advisory board of Numera, Twin Health, L-Neutra, he is a share-holder of Healthimation, and he has received consultation honoraria from Abbott Nutrition, Sanofi, and Merck Serono. The remaining authors declare that the research was conducted in the absence of any commercial or financial relationships that could be construed as a potential conflict of interest.

## Publisher's Note

All claims expressed in this article are solely those of the authors and do not necessarily represent those of their affiliated organizations, or those of the publisher, the editors and the reviewers. Any product that may be evaluated in this article, or claim that may be made by its manufacturer, is not guaranteed or endorsed by the publisher.
