# Supplementary material for: The Transcultural Diabetes Nutrition Algorithm: A Middle Eastern Version
Source: Front Nutr. 2022 Jun 13;9:899393. doi: 10.3389/fnut.2022.899393 (PMC9235861; doi:10.3389/fnut.2022.899393)
Supplement: Supplementary file 1 [file Data_Sheet_1.pdf]

Supplementary Material

Transcultural Diabetes Nutrition Algorithm: a Middle Eastern Version: Summary of Figures and Tables for Clinical Practice

This *Supplementary Material* has been compiled to support the full-length paper by Hamdy et al [1]. It is intended as a convenient and user-friendly summary for clinicians who treat people with prediabetes and Type 2 diabetes in the Middle East. To meet this aim, the summary includes figures and tables from the text body and some supplementary tables.

Figure 1. Middle East Transcultural Diabetes Nutrition Algorithm for Prediabetes and Type 2 Diabetes. Adapted for the Middle East from Mechanick et al [2].

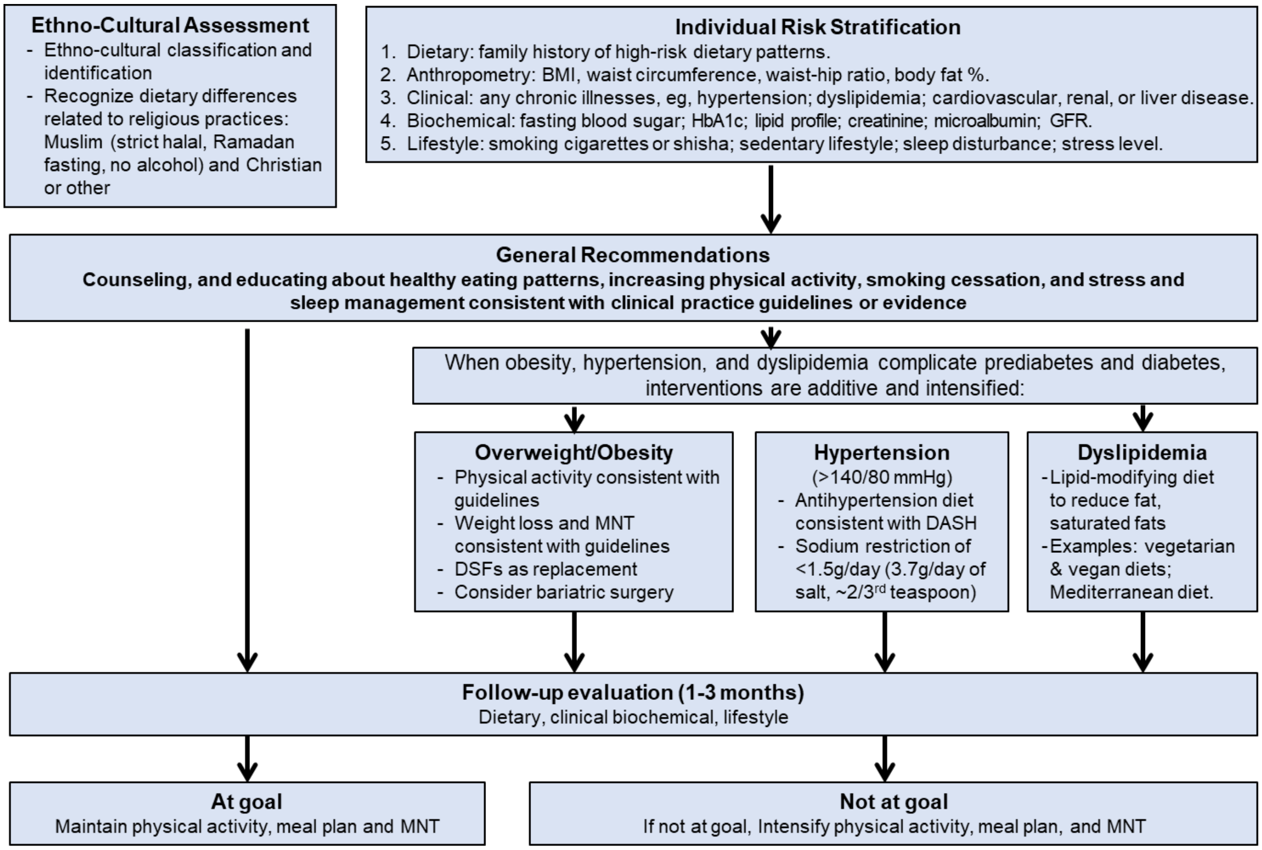

Figure 2. A Healthy Eating Plate to Guide Dietary Intake

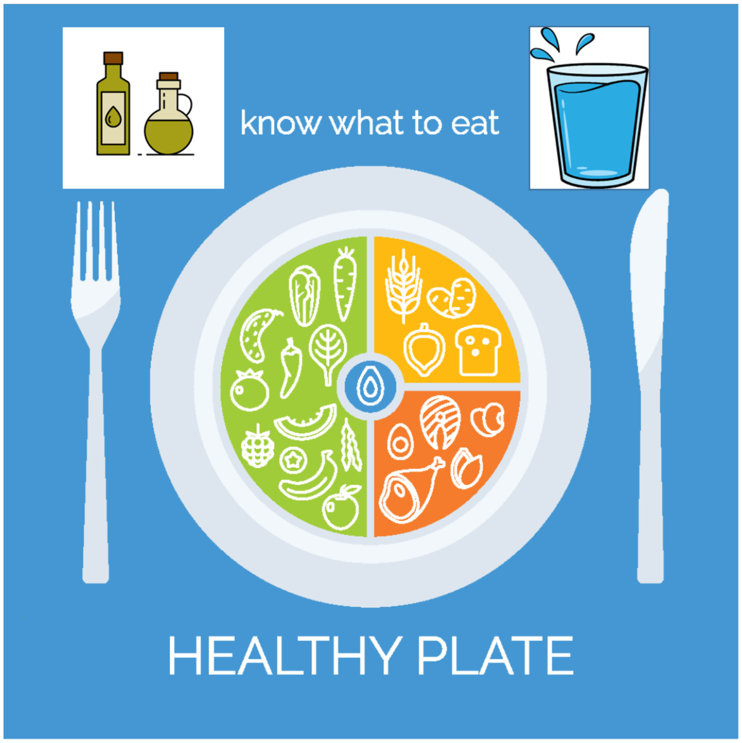

Diabetes, Nutrition, and Ramadan

Eat a varied and balanced diet with plenty of vegetables and fruits—up to 50% of your plateful.

Carbohydrate

- **Eat carbohydrate foods with low glycemic index**
- **Include fiber-rich carbohydrates**

Protein

- **20-30% of total caloric intake**
- **Fish, skinless poultry, dairy, nuts, seeds, and legumes**

Fat

- **Less than 35% of total caloric intake**
- **Healthy fats (olive and canola) are recommended**
- **Foods high in saturated fats or trans-fats are not recommended**

Tips for Ramadan

- **Consult your doctor if you plan to fast; learn your risk.**
- **Drink plenty of water during non-fasting period to avoid dehydration during fasting.**

Figure 3. Arab Food Dome [3,4]

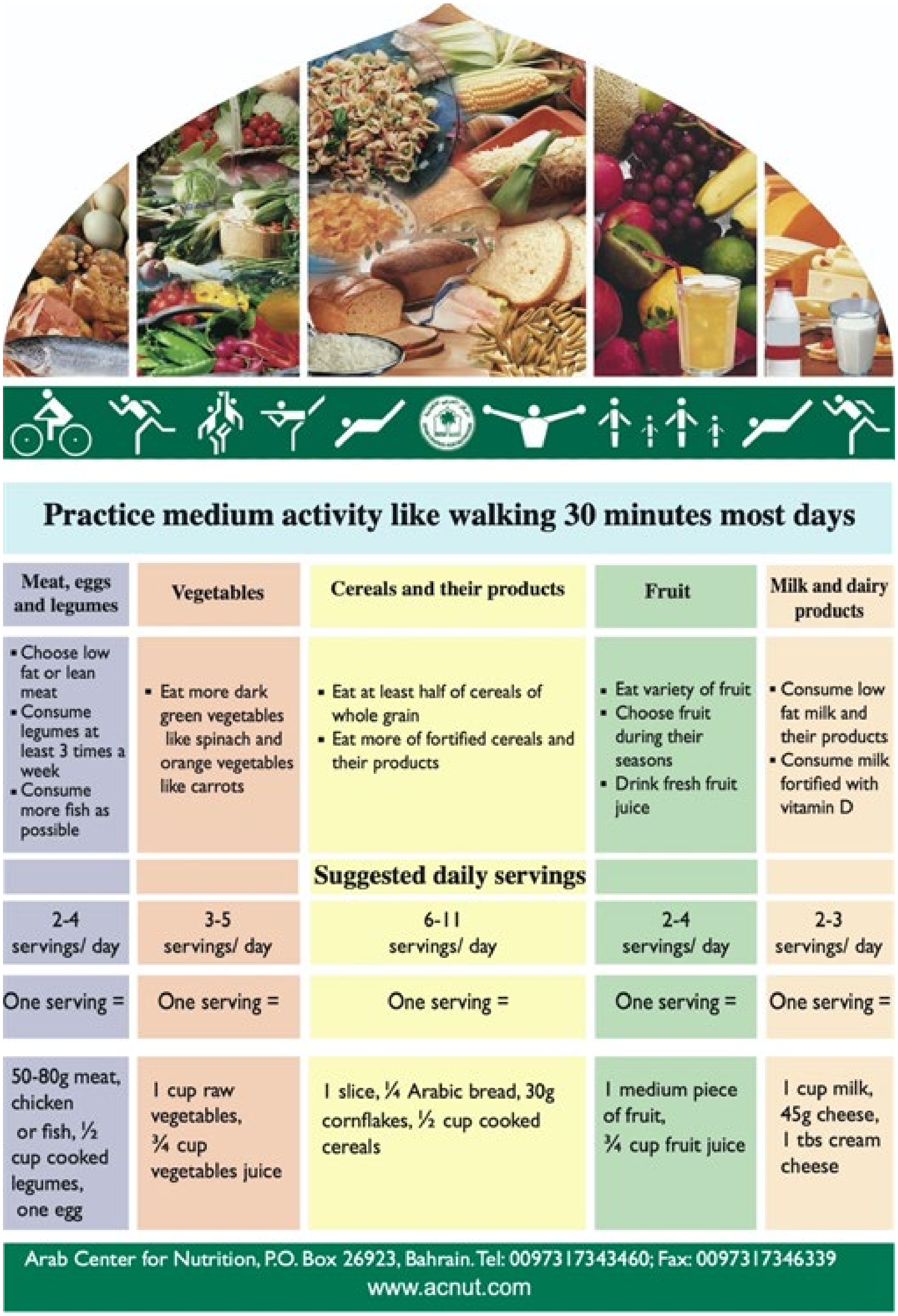

Table 1. Diagnostic criteria for prediabetes and diabetes [5]

	PREDIABETES	DIABETES
**FPG (MG/DL)**	100 – 125	≥ 126
**2H OGTT (MG/DL)**	140 –199	≥ 200
**CASUAL PG (MG/DL)**	< 200	≥ 200
**A1C**	5.7 – 6.4%	≥ 6.5%

Definitions: FPG, fasting plasma glucose; 2h OGTT, 2-hour oral glucose tolerance test; PG, plasma glucose; A1C, hemoglobin A1C.

Table 2. Body composition (BMI and WC) and disease risk (type 2 diabetes, hypertension, and cardiovascular disease)[6-9]

			**Waist Circumference and Disease Risk***	
	**BMI, kg/m^2**	**Obesity Class**	**Men ≤ 102cm Women ≤ 88 cm**	**Men > 102cm Women > 88 cm**
Underweight	<18.5			
Normal weight	18.5–24.9			
Overweight	25.0–29.9		Increased	High
Obese	30.0–34.9	I	High	Very high
	35.0–39.9	II	Very high	Very high
Extremely obese	≥40	III	Extremely high	Extremely high

Definitions: BMI, body mass index; WC, waist circumference.

Table 3. Antihypertensive DASH dietary goals for the Middle East (2,000 calories/day eating plan) [10-12]

Nutrient	Recommendations
**Macronutrients**	
**Protein**	**18% of total calories**
**Carbohydrate**	**55% of total calories**
Fiber	30 g/day
**Total fat**	**27% of total calories**
Saturated fat	6% of total calories
Cholesterol	150 mg/day
**Micronutrients**	
Sodium	1500 mg/day
Potassium	4700 mg/day
Calcium	1250 mg/day
Magnesium	500 mg/day

Definitions: DASH, Dietary Approaches to Stop Hypertension.

Table 4. Glycemic index (GI) values of some common Middle Eastern foods by food group*
GI values below 55 are low, 56-69 values are moderate, and values >70 are high.

Fruits and vegetables*		Meat, fish, dairy*		Carbohydrates*	
**Food**	**GI**	**Food**	**GI**	**Food**	**GI**
Carrots, raw	35	Chickpeas	10	Khameer bread	47
Carrots, boiled	39	Yogurt, plain Greek	11	Multigrain bread	53
Dates, Khalas	36	Feta cheese	27	Chebab bread	54
Apple	38	Lentils	29	Brown rice	55
Arnana	52	Milk, fat-free	32	Oatmeal	58
Grapes	59	Yogurt, sweetened fruit	36	White rice, boiled	64
Sweet corn	60	Milk, full fat	41	Couscous	65
Banana	62	Burghol	48	Arabic pita bread	67
Watermelon	72	Chicken, biryani	52	Popcorn, air popped	72
Dates, Sellaj	75	Fish machboos	60	Fendal, sweet potato	74
		Beef thareed	74	Regag bread	76
		Cheese fatayer	80	Muhalla bread	77
				Tannour white bread	81
				Corn flakes	81
				Awama	81
				White potato, boiled	82
				Sharia (vermicelli)	83
				White basmati rice	84

The GI formula is (iAUC of a test food divided by iAUC of a reference food) × 100. Abbreviations: iAUC – incremental area under curve; GI - glycemic index. See references [13-16].

Table 5. Glycemic index (GI) of common Middle Eastern dishes [13,15,16]

Food	GI	Food	GI
Fatayer cheese (Qatar)	80.3	Stuffed cabbage with rice & meat	67.9
Fatayer zaatar (Qatar)	79.6	Green beans in oil	12.8
Fatayer spinach (Qatar)	77.6	Baked muttabaq (Saudi Arabia)	56
Machbous fish (UAE)	60	Harees (Saudi Arabia)	52
Burghol with tomatoes (Lebanon)	50.1		
Harees (UAE)	42		
Thareed beef (UAE)	74	***Desserts:***	
Biryani chicken (UAE)	52	Awama (fried doughnuts Qatar)	81.4
Arseyah (basmati rice w/chicken)	72	Qurs Aquili (Qatar)	83.2
Khabisa (semolina with cardamon)	67	Muhalabia (milk with starch & sugar)	83.2
Pizza	56	Riz bi halib (milk with rice & sugar)	56.8
Sambosa vegetable	60	Batheetha (khalas date paste)	59
Red beans with white bread	61	Kanfaroosh (doughnut cake)	45
Mjadara (lentils & rice, Lebanon)	24	Balaleet	63
Stuffed grape leaves	30	Shearia (Qatar)	83
Moroccan couscous	58	Dates with Arabic coffee	63
Kibbeh saynyeh	61	Dates with sour milk or yogurt	29

GI, glycemic index formula is (iAUC of a test food divided by iAUC of a reference food) × 100.

Table 6. Diabetes-specific formulas (DSFs) for prediabetes and diabetes [2]

<table>
<tr><th>BMI classification</th><th colspan="2">DSF Recommendation</th></tr>
<tr><td>Overweight
or obese</td><td colspan="2">• Use 2 to 3 units per day of a diabetes-specific nutrition formula[a] as part of a reduced calorie meal plan, as a calorie replacement for a meal, partial meal, or a snack.
• Daily calorie goals from diabetes-specific nutrition formulas and other healthy dietary sources:
Women = 1200 to 1500 calories
Men = 1500 to 1800 calories</td></tr>
<tr><td rowspan="2">Normal weight</td><td>Uncontrolled diabetes, A1c > 7%</td><td>1 to 2 units per day of diabetes-specific nutrition formula incorporated into a meal plan, as a calorie replacement for a meal, partial meal, or a snack.</td></tr>
<tr><td>Controlled diabetes, A1c ≤ 7%</td><td>The use of diabetes-specific nutrition formulas should be based on individual patient needs and clinical judgment of the healthcare professional[b]</td></tr>
<tr><td>Underweight</td><td colspan="2">1 to 3 units per day of diabetes-specific nutrition supplements per clinical judgment based on desired rate of weight gain and clinical tolerance[c]</td></tr>
</table>

[a]DSFs are complete and balanced products with at least 200 calories per serving used as part of a meal plan to help control calorie intake and achieve glycemic control.
[b]Meal and snack replacements are nutritional products used to replace dietary calories.
[c]To avoid hypoglycemia or postprandial hyperglycemia, individuals who may have muscle mass and/or function loss and/or micronutrient deficiency may benefit from a nutrition supplement. Individuals who need support with weight maintenance and/or a healthy meal plan could benefit from meal replacement.

Table 1 Supplemental. Nutrition guidelines for type 2 diabetes (T2D) in the Middle East

	**Guidelines**
1. Weight management for patients with overweight or obesity	• Prediabetes: people with prediabetes should lose 7–10% of their body weight to prevent progression to T2D [17,18].<br>• T2D: in conjunction with lifestyle therapy, medication-assisted weight loss can be considered for people at risk for T2D when needed to achieve and sustain a 7–10% weight loss [18].
2. Macronutrient distribution	• Evidence suggests that there is not an ideal percentage of calories from carbohydrate, protein, and fat for people with diabetes.<br>• Macronutrient distribution should be based on an individualized assessment of current eating patterns, preferences, and metabolic goals.
3. Dietary guidelines for Arab countries	• In absence of dietary guidelines specific to Arab people, regional Health Institutes primarily used Western dietary guidelines T2D<br>• In 2011, the Arab Centers for Nutrition established the 'Food Dome', dietary guidelines to prevent disease for the Arab region [4].
4. Mediterranean-style eating pattern	This dietary pattern can improve both glycemic management and blood lipids. Characteristics of these diets include [19]:<br>• Plant-based: abundant in fruits and vegetables; breads and other forms of cereals; and beans, nuts, and seeds.<br>• Minimally processed: locally grown, seasonally fresh foods.<br>• Limited sweets: fresh fruits are the typical daily dessert, with sweets based on nuts and made with olive oil.<br>• High-quality fats: olive oil is the primary source of fat, and total intake is moderate (30%) to high (40%) of total energy intake.<br>• Low to moderate dairy intake: mainly cheese and yogurt.<br>• Protein: red meats and eggs are consumed in small amounts and with low frequency; seafood intake varies, with moderate amounts of fish.<br>• Herbs and spices: used instead of salt to add flavor to foods.
5. Fiber intake	14g of fiber per 1000 kcals [20,21]
6. Restrictions	Minimize processed foods [22]

Table 2 Supplemental. Physical activity guidelines for the Middle East [23]

	**Guidelines**
**1. Reducing sedentary time**	• All adults, and particularly those with type 2 diabetes, should decrease the amount of time spent in daily sedentary behavior.<br>• Prolonged sitting should be interrupted with bouts of light activity every 30 min for blood glucose benefits, at least in adults with type 2 diabetes.<br>• The above two recommendations are additional to, and not a replacement for, increased structured exercise and incidental movement.
**2. Physical activity and Type 2 diabetes**	• Daily exercise, or at least not allowing more than 2 days to elapse between exercise sessions, is recommended to enhance insulin action.<br>• Adults with type 2 diabetes should ideally perform both aerobic and resistance exercise training for optimal glycemic and health outcomes.<br>• Children and adolescents with type 2 diabetes should be encouraged to meet the same physical activity goals set for youth in general.<br>• Structured lifestyle interventions that include at least 150 min/week of physical activity and dietary changes resulting in weight loss of 5%–7% are recommended to prevent or delay the onset of type 2 diabetes in populations at high risk and with prediabetes.
**3. Hydration and heat concerns**	• Hyperglycemia increases risk through dehydration caused by osmotic diuresis, and some medications that lower blood pressure may also impact hydration and electrolyte balance.<br>• Older adults with diabetes or anyone with autonomic neuropathy, cardiovascular complications, or pulmonary disease should avoid exercising outdoors on very hot and/or humid days.

Table 3 Supplemental. Bariatric Surgery Criteria in T2D [24-27]

Requirements for each bariatric surgery patient:

- Has attempted lifestyle modification but failed to achieve and sustain weight loss
- Can tolerate surgical risks
- Is committed to treatment and long-term follow-up
- Has accepted the required lifestyle changes

BMI Category	Recommendations
BMI ≥40 kg/m^2	Bariatric surgery is recommended for these high BMI individuals, i.e., about 45 kg overweight for men or 36 kg overweight for women.
MI 35-39.9 kg/m^2	Bariatric surgery is recommended for those with a serious obesity-related comorbidity, such as T2D, coronary heart disease, or severe sleep apnea.
BMI 30-34.9 kg/m^2	Consider bariatric surgery as an alternative treatment option for those with special circumstances:<br>• When diabetes is not adequately controlled by an optimal medical regimen, especially when there are risks for cardiovascular disease.<br>• To achieve at least short-term weight reduction in patients with T2D (laparoscopic-assisted gastric banding, sleeve gastrectomy, or Roux-en-Y gastric bypass).

References

1. Hamdy, O.; Al Sifri, S.; Hassanein, M.; Al Dawish, M.; Al-Dahash, R.; Alawadi, F.; Jarrah, N.; Ballout, H.; Amin, A. The transcultural Diabetes Nutrition Algorithm: a Middle Eastern version. *Nutrients* **2022**.
2. Mechanick, J.I.; Marchetti, A.E.; Apovian, C.; Benchimol, A.K.; Bisschop, P.H.; Bolio-Galvis, A.; Hegazi, R.A.; Jenkins, D.; Mendoza, E.; Sanz, M.L., et al. Diabetes-specific nutrition algorithm: a transcultural program to optimize diabetes and prediabetes care. *Curr Diab Rep* **2012**, *12*, 180-194, doi:10.1007/s11892-012-0253-z.
3. Arab Center for Nutrition. Food Dome: Dietary guidelines for Arab countries. Available online: https://www.acnut.com/v/images/stories/pdf/qeben.pdf (accessed on 23 Feb 2021 2021).
4. Musaiger, A.O.; Arab Centers for Nutrition. The Food Dome: dietary guidelines for Arab countries. *Nutr Hosp* **2012**, *27*, 109-115, doi:10.1590/S0212-16112012000100012.
5. American Diabetes Association. 2. Classification and diagnosis of diabetes: standards of medical care in diabetes-2020. *Diabetes Care* **2020**, *43*, S14-S31, doi:10.2337/dc20-S002.
6. World Health Organization. *Waist circumference and waist–hip ratio: report of a WHO expert consultation*; Geneva, Switzerland, 8-11 December, 2008.
7. American Diabetes, A.; Bantle, J.P.; Wylie-Rosett, J.; Albright, A.L.; Apovian, C.M.; Clark, N.G.; Franz, M.J.; Hoogwerf, B.J.; Lichtenstein, A.H.; Mayer-Davis, E., et al. Nutrition recommendations and interventions for diabetes: a position statement of the American Diabetes Association. *Diabetes Care* **2008**, *31 Suppl 1*, S61-78, doi:10.2337/dc08-S061.
8. Purnell, J. Definitions, classification, and epidemiology of obesity. In *Endotext [Internet]*, Feingold, K.R., Anawalt, B., Boyce, A., Eds. MDText.com, Inc: South Dartmouth, MA, 2018.
9. National Heart Lung and Blood Institute. *Clinical guidelines on the identification, evaluation, and treatment of overweight and obesity in adults—the evidence report*; 98-4083; National Institutes of Health: 1998.
10. Campbell, A.P. DASH eating plan: an eating pattern for diabetes management. *Diabetes Spectr* **2017**, *30*, 76-81, doi:10.2337/ds16-0084.
11. National Heart Lung and Blood Institute. DASH eating plan. Available online: https://www.nhlbi.nih.gov/health-topics/dash-eating-plan (accessed on 10 Feb 2021 2021).
12. U.S. Department of Health and Human Services; National Institutes of Health; National Heart Lung and Blood Institute. *Your guide to lowering blood pressure with DASH*; 06-5834; NIH: 2015.
13. Al-Mssallem, M. The association between the glycaemic index of some traditional Saudi foods and the prevalence of diabetes in Saudi Arabia: a review article. *J Diabetes Metab* **2014**, *5*, 452, doi:10.4172/2155-6156.1000452.
14. AlGeffari, M.A.; Almogbel, E.S.; Alhomaidan, H.T.; El-Mergawi, R.; Barrimah, I.A. Glycemic indices, glycemic load and glycemic response for seventeen varieties of dates grown in Saudi Arabia. *Ann Saudi Med* **2016**, *36*, 397-403, doi:10.5144/0256-4947.2016.397.
15. Ali, A.; Al-Hakmani, M.; Waly, M.; Essa, M. Glycemic index of commonly consumed snack foods in Oman. *Int J Nutr Pharmacol Neurol Dis* **2020**, *10*, 50-56.
16. Atkinson, F.S.; Foster-Powell, K.; Brand-Miller, J.C. International tables of glycemic index and glycemic load values: 2008. *Diabetes Care* **2008**, *31*, 2281-2283, doi:10.2337/dc08-1239.
17. Hamdy, O.; Mottalib, A.; Morsi, A.; El-Sayed, N.; Goebel-Fabbri, A.; Arathuzik, G.; Shahar, J.; Kirpitch, A.; Zrebiec, J. Long-term effect of intensive lifestyle intervention on cardiovascular risk factors in patients with diabetes in real-world clinical practice: a 5-year

longitudinal study. *BMJ Open Diabetes Res Care* **2017**, *5*, e000259, doi:10.1136/bmjdrc-2016-000259.
18. American Diabetes Association. Standards of medical care in diabetes-2020. *Diabetes Care* **2020**, *43*, S1-S212.
19. Boucher, J.L. Mediterranean eating pattern. *Diabetes Spectr* **2017**, *30*, 72-76, doi:10.2337/ds16-0074.
20. Academy of Nutrition and Dietetics. Fiber. Available online: https://www.eatright.org/food/vitamins-and-supplements/nutrient-rich-foods/fiber (accessed on 16 Apr 2021).
21. Evert, A.B.; Dennison, M.; Gardner, C.D.; Garvey, W.T.; Lau, K.H.K.; MacLeod, J.; Mitri, J.; Pereira, R.F.; Rawlings, K.; Robinson, S., et al. Nutrition therapy for adults with diabetes or prediabetes: a consensus report. *Diabetes Care* **2019**, *42*, 731-754, doi:10.2337/dci19-0014.
22. Srour, B.; Fezeu, L.K.; Kesse-Guyot, E.; Alles, B.; Debras, C.; Druesne-Pecollo, N.; Chazelas, E.; Deschasaux, M.; Hercberg, S.; Galan, P., et al. Ultraprocessed food consumption and risk of type 2 diabetes among participants of the NutriNet-Sante prospective cohort. *JAMA Intern Med* **2020**, *180*, 283-291, doi:10.1001/jamainternmed.2019.5942.
23. Colberg, S.R.; Sigal, R.J.; Yardley, J.E.; Riddell, M.C.; Dunstan, D.W.; Dempsey, P.C.; Horton, E.S.; Castorino, K.; Tate, D.F. Physical activity/exercise and diabetes: a position statement of the American Diabetes Association. *Diabetes Care* **2016**, *39*, 2065-2079, doi:10.2337/dc16-1728.
24. Cummings, D.E.; Rubino, F. Metabolic surgery for the treatment of type 2 diabetes in obese individuals. *Diabetologia* **2018**, *61*, 257-264, doi:10.1007/s00125-017-4513-y.
25. NIDDK. Weight-loss (bariatric) surgery. Available online: https://www.niddk.nih.gov/health-information/weight-management/bariatric-surgery (accessed on Mar 19 2021
26. Rubino, F.; Nathan, D.M.; Eckel, R.H.; Schauer, P.R.; Alberti, K.G.; Zimmet, P.Z.; Del Prato, S.; Ji, L.; Sadikot, S.M.; Herman, W.H., et al. Metabolic surgery in the treatment algorithm for type 2 diabetes: a joint statement by international diabetes organizations. *Diabetes Care* **2016**, *39*, 861-877, doi:10.2337/dc16-0236.
27. Arterburn, D.E.; Telem, D.A.; Kushner, R.F.; Courcoulas, A.P. Benefits and risks of bariatric surgery in adults: a review. *JAMA* **2020**, *324*, 879-887, doi:10.1001/jama.2020.12567.
